# RETRACTION: VEGF Induce Vasculogenic Mimicry of Choroidal Melanoma through the PI3k Signal Pathway

**DOI:** 10.1155/bmri/9763103

**Published:** 2026-01-14

**Authors:** BioMed Research International

RETRACTION: X. Xu, Y. Zong, Y. Gao, X. Sun, H. Zhao, W. Luo, and S. Jia, “VEGF Induce Vasculogenic Mimicry of Choroidal Melanoma through the PI3k Signal Pathway,” *BioMed Research International* 2019, no. 1 (2019): 1–13, https://doi.org/10.1155/2019/3909102.

The above article, published online on 11 July 2019 in Wiley Online Library (http://wileyonlinelibrary.com), has been retracted by agreement between the journal Editor, Raj P. Kandpal and John Wiley & Sons Ltd.

The retraction has been agreed upon following an investigation into concerns raised by a third party and Pubpeer [1] regarding image duplication.

Image duplication was identified with Figure 7a and three other unrelated articles, which pre‐ and post‐date the article in question, sharing no authors or affiliations.

• Figure 7a, image ‘OCM‐1 (COCl_2_)’ matches the image presented in Di et al 2017 [2], Figure 3c, TPC‐1/Control.
• Figure 7a, image ‘OCM‐1’ matches the image presented in Su et al 2020 [3], Figure 6b, pcDNA3.1.
• Figure 7a, image ‘siVEGF’ matches the image presented in Chen et al 2022 [4], Figure 4b, HSO/miR‐135a + SMAD2.
• Figure 7a, image ‘OCM‐1 (COCl_2_)’ matches the image presented in Chen et al 2022 [4], Figure 4b, MG63/miR‐135a + vector.

The authors’ response to questions regarding the article’s images was insufficient, adding further to concerns over the article.

The authors agree with the retraction.

## References

[1] Hoya camphorifolia , VEGF Induce Vasculogenic Mimicry of Choroidal Melanoma Through the PI3k Signal Pathway, *PubPeer*, 2023, https://pubpeer.com/publications/5A3CD8386F0CC70BFBE0C06A414EB2. 10.1155/2019/3909102 PMC6657640 31380420

[2] Di W. , Li Q. , Shen W. , Guo H. , and Zhao S. , The Long Non-Coding RNA HOTAIR Promotes Thyroid Cancer Cell Growth, Invasion and Migration Through the miR-1-CCND2 Axis, American Journal of Cancer Research. (2017) 7, no. 6, 1298–1309, 2017. 28670492 PMC5489779

[3] Su X. , Gao C. , Feng X. , and Jiang M. , [Retracted] miR-613 Suppresses Migration and Invasion in Esophageal Squamous Cell Carcinoma Via the Targeting of G6PD, Experimental and Therapeutic Medicine. (2023) 26, no. 6, 10.3892/etm.2023.12246, 37928508. PMC10623213 37928508

[4] Chen Y. , Cai B. , Lian X. , Xu J. , and Zhang T. , MiR-135a Targets SMAD2 to Promote Osteosarcoma Proliferation and Migration, Journal of Oncology. (2022) 2022, no. 1, 1–9, 10.1155/2022/3037348. PMC9020941 35466322

